# The interplay between mindfulness, depression, stress and academic performance in medical students: A Saudi perspective

**DOI:** 10.1371/journal.pone.0231088

**Published:** 2020-04-03

**Authors:** Ahmed M. Alzahrani, Ahmed Hakami, Ahmad AlHadi, Mohammed A. Batais, Abdullah A. Alrasheed, Turky H. Almigbal

**Affiliations:** 1 Department of Family and Community Medicine, College of Medicine, King Saud University, Riyadh, Saudi Arabia; 2 Department of Pathology, College of Medicine, King Saud University, Riyadh, Saudi Arabia; 3 Riyadh Regional Laboratory, Ministry of Health, Riyadh, Saudi Arabia; 4 Department of Psychiatry, College of Medicine, King Saud University, Riyadh, Saudi Arabia; 5 SABIC Psychological Health Research & Applications Chair, College of Medicine, King Saud University, Riyadh, Saudi Arabia; 6 Alfarabi College of Medicine, Alfarabi Colleges, Riyadh, Saudi Arabia; King Fahd University of Petroleum & Minerals, SAUDI ARABIA

## Abstract

There is a growing body of research that shows a significant association between mindfulness and mental health. However, studies on Saudi populations are still in their infancy. Mindfulness is a personal tendency to focus on the present time in a non-judgmental manner, including the interior and exterior experience of feelings and events. The first aim of this study is to examine the relationship between mindfulness, stress, depression, and academic performance in a sample of medical students from King Saud University. The second aim is to explore the potential moderation effects of mindfulness on the impact of stress on academic performance and depression in the study population. This cross-sectional study examined 289 medical students who were selected by a stratified random sampling technique and completed validated online questionnaires measuring mindfulness, stress, and depression. The data were analyzed using SAS version 9.2, and R software was used for graphs. Correlation analysis showed that mindfulness is inversely associated with depression and stress, but not with academic performance. Furthermore, multiple logistic regression showed that mindfulness can predict both depression and stress. We also found that two subscales of mindfulness can moderate the relation between stress and depression: non-judging of inner experience and describing. The findings suggest that a higher mindfulness score is associated with lower depression and stress levels and could buffer against depression in a stressful environment. There is a need for further research to investigate the relation of mindfulness with positive psychological outcomes, as well as experimental trials to examine the efficacy of mindfulness training on improving mental wellbeing in our community.

## Introduction

Mental distress is a widely acknowledged subject in medical education. Past research indicates that medical students are subjected to high levels of stress and depression [1]. A meta-analysis concluded that the global prevalence of depression among medical students is 28%, and first-year medical students have the highest rates [2]. An Eastern study showed a stress prevalence of 29.6% in 761 medical students at Universiti Sains Malaysia [3]. These findings are fairly concordant with our local studies. Regionally, a cross-sectional study that was conducted in King Saud University (KSU) Medical School revealed that the estimated prevalence of depression among medical students was 46.2% [4]. Another local study screened KSU medical students for stress and concluded that 61% of them were under some degree of stress, but 25% had severe stress, and females scored significantly higher levels than males [5].

The impact of this problem has also been studied in different aspects. In relation to future medical careers, evidence has suggested that psychological distress during medical school could anticipate problems later in clinical practice [6, 7], which could affect patient care [8]. Furthermore, medical professionals often do not seek help from mental health experts when needed [9], and students may also show the same behavior [2, 10]. In addition, the academic performance of medical students might also be affected by mental distress [11–13], which could result in them quitting medical school [14, 15].

Researchers have investigated these issues with great emphasis on the role of mindfulness [16–19]. In Western populations, it has been shown that a higher level of mindfulness is negatively associated with stress [19] and depression [17]. As for academic performance, mindfulness also has a positive effect on grade point average (GPA) [20]. The most cited definition of mindfulness is “the awareness that emerges through paying attention on purpose, in the present moment, and nonjudgmentally to the unfolding of experience moment by moment” [21]. As such, mindfulness encompasses a set of meditation skills that augment momentary mental presence [22]. It is theoretically accepted that mindfulness is an inherent humane trait and one that can be developed with training [23].

Studies about mindfulness and its association with mental health on the Saudi population are still in their infancy. To our knowledge, publications in this area of interest are lacking in our community. In the current study, we aimed to explore the relationship between mindfulness as a human capacity, depression, stress, and GPA among KSU medical students. Furthermore, we also explore the potential moderation effect of mindfulness and its subscales on the impacts of stress on academic performance and depression in the study population. We hypothesized that mindfulness would have an inverse relationship with depression and stress and a positive association with higher GPA. We also hypothesized that mindfulness plays a moderating role in the impact of stress on depression and GPA.

## Methodology

### Participant enrolment

This cross-sectional study was conducted in the College of Medicine of KSU in Riyadh, Saudi Arabia. The KSU medical school involves five years of study and one year of internship. The total number of medical students is 1408 (823 are males). From March to April 2018, a stratified random sampling technique was employed for data collection to include both genders from all academic years of KSU medical school. A list of students was obtained from the student affairs department, and a random sequence of numbers was generated by Microsoft Excel. A group of 9 medical students volunteered to communicate with the randomly selected participants and to send them the study summary with a participation consent form and online questionnaires by e-mail.

To achieve the study objectives, the estimated sample size was 228. Raosoft Sample Size Calculator was used to calculate the sample size based on the parameters estimated for the study population [24]. We chose a 5% margin of error with a confidence interval of 90%. The sample size was increased by 25% to account for non-responses. All active medical students in KSU in March to April 2018 were eligible to participate in the current study, and there were no exclusion criteria.

### Study variables

Depression, stress, and last-semester GPA were the dependent variables, while demographics (age, gender, undergraduate year, and history of meditation) and mindfulness were the independent variables.

### Measures

In the current study, the 39-item Five-Facet Mindfulness Questionnaire (FFMQ) was used to measure the tendency to be mindful in daily life. The five facets are observing, describing, acting with awareness, non-judging of inner experience, and non-reactivity to inner experience. It is designed with a five-point Likert-scale ranging from 1 (“never or very rarely true”) to 5 (“very often or always true”), with a higher score indicating better mindfulness skills. The alpha coefficients for all facets were adequate to good (range: 0.72 to 0.92) [25]. To measure depression and stress, both the Patient Health Questionnaire 9 (PHQ-9) and Perceived Stress Scale (PSS) were used, which are well-known and valid questionnaires [26, 27]. Academic performance was assessed using the students’ last-semester GPA. Age, gender, undergraduate year, and history of meditation (meditating vs. non-meditating) were the demographic variables. No permissions were required to use these questionnaires.

### Data analysis

Data are presented as means and standard deviations (SD) for continuous variables and as frequencies and percentages for categorical ones. Correlations between dependent and independent variables were assessed using Pearson’s correlation coefficient. We used multiple logistic regression to identify independent predictors for stress and depression. Least-square means of interactions were used to check whether mindfulness subscales moderate the relation between stress and depression. All statistical analyses were performed using SAS version 9.2 (SAS Institute, Inc., Cary, NC), and graphs were obtained using R (R foundation for Statistical Computing, Vienna, Austria).

### Ethical considerations

This study was ethically approved by the Institutional Review Board of the College of Medicine at KSU (project number: E-18-3024). Consent was obtained from the participants, who were assured that their participation would be voluntary and anonymous. Data were collected through online surveys and stored in the principal investigator’s personal computer, which has no public access and is password secured.

## Results

### Demographics and clinical characteristics

Our sample of 289 medical students (response rate of 85%) spanned all academic years, and their demographic information is presented in Table 1. There was almost equal gender representation, and the mean age was 21.5 years (SD 1.6 years). More than 90% of the sample had very good scores and excellent GPA. Moderate and severe levels of stress were seen in 65.1% and 15.2% of the participants, respectively. The percentages of moderate, moderately severe, and severe depression were 20.4%, 9.3%, and 9%, respectively. The mean score of mindfulness was 118.84 (SD 15.6), and the mean subscale mean scores were 23.95 (SD 5.65) for observing, 25.32 (SD 6.16) for describing, 23.45 (SD 6.93) for non-judging of inner experience, 20.28 (SD 4.16) for non-reactivity to inner experience, and 25.84 (SD 6.56) for acting with awareness.

**Table 1:** Demographics and clinical characteristics.

Variable	Level	N = 289	%
Age	Mean	21.50	-
	Std Dev	1.60	-
Gender	Male	140.00	48.4
	Female	149.00	51.6
Academic year	1st year	65.00	22.5
	2nd year	75.00	26.0
	3rd year	40.00	13.8
	4th year	49.00	17.0
	5th year	60.00	20.8
GPA	Good	22.00	7.6
	Very Good	132.00	45.7
	Excellent	135.00	46.7
Personal history of meditation	Meditating	79.00	27.3
	Not meditating	210.00	72.7
Stress	Low	57.00	19.7
	Moderate	188.00	65.1
	High	44.00	15.2
Depression	No	81.00	28.0
	Mild	96.00	33.2
	Moderate	59.00	20.4
	Moderately Severe	27.00	9.3
	Severe	26.00	9.0
Mindfulness	Mean	118.84	-
	Std Dev	15.60	-
**The five subscales of mindfulness**			
Observing	Mean	23.95	-
	Std Dev	5.65	-
Describing	Mean	25.32	-
	Std Dev	6.16	-
Non-judging of inner experience	Mean	23.45	-
	Std Dev	6.93	-
Non-reactivity to inner experience	Mean	20.28	-
	Std Dev	4.16	-
Acting with awareness	Mean	25.84	-
	Std Dev	6.56	-

### Association of mindfulness with depression, stress, and academic performance

**Fig 1:**
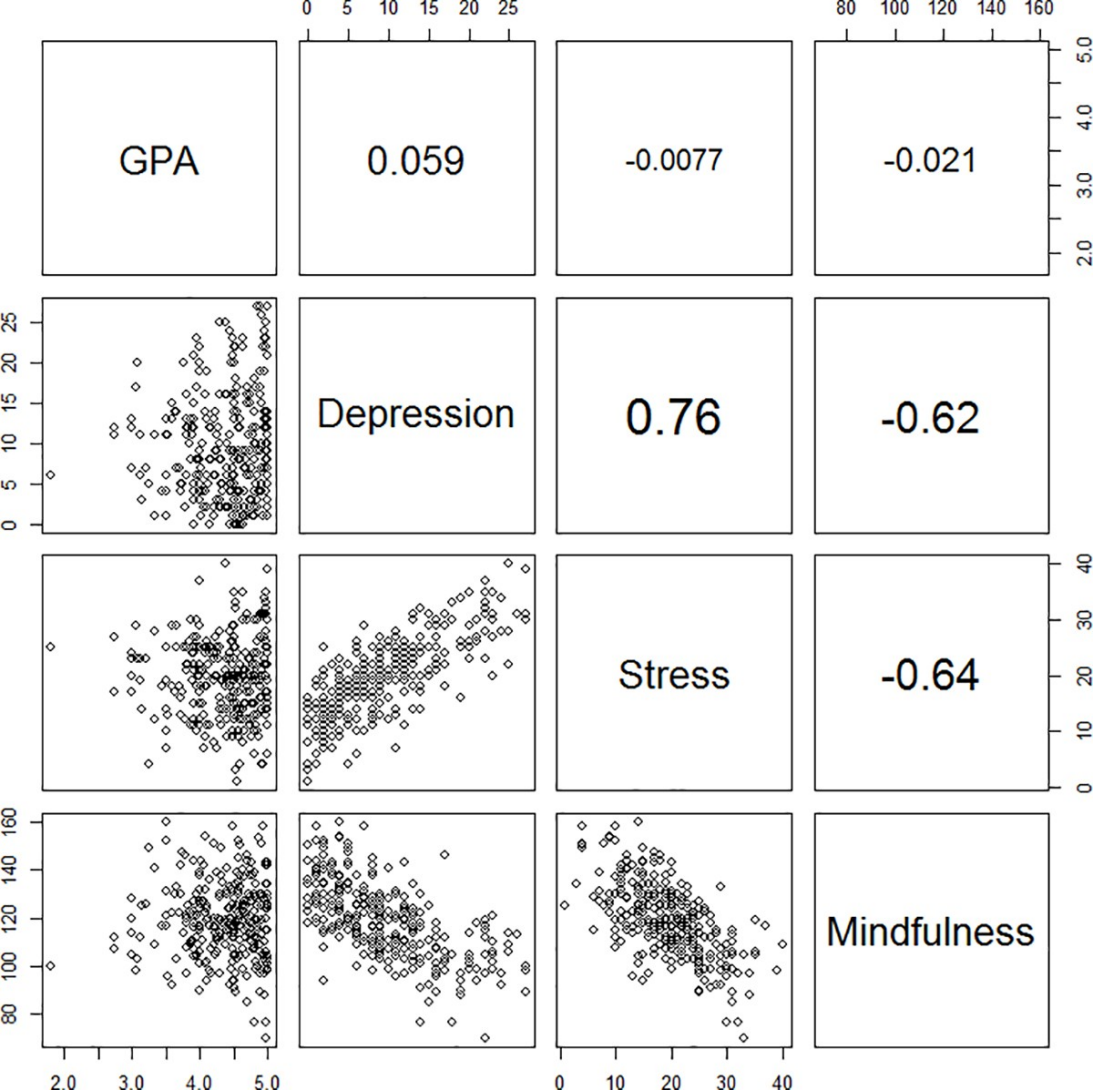
Scatter plot chart showing the linear correlation of mindfulness with other variables.

The correlation analysis revealed a statistically significant inverse relationship between mindfulness and both stress and depression (Fig 1, Table 2). However, last-semester GPA did not. Multiple logistic regression analysis was performed to predict the effect of mindfulness as a continuous variable on stress and depression as categorical variables (yes or no), as shown in Tables 3 and 4. The analysis showed that an increase of one unit in the mindfulness score corresponded to a decrease in the chance of being stressed by 5% (OR 0.95, CI (0.93–0.98), P < 0.002) and the probability of being depressed by 7% (OR 0.93, CI (0.90–0.96), P < 0.001).

**Table 2:** Correlation coefficient of mindfulness with other variables.

	Mindfulness		
Variable	N	Pearson CC	Pearson P-value
Last-semester GPA	289	-0.021	0.725
Depression	289	-0.619	**<0.001**
Stress	289	-0.640	**<0.001**

**Table 3:** Multiple logistic regression for predictors of stress.

		Response stress = yes			
Covariate	Level	Odds Ratio	95%CI Low	95%CI Up	OR P-value
Age (years)	One year increase	1.12	0.70	1.81	0.627
Gender	male	0.49	0.22	1.07	0.073
Academic year	1st year	2.46	0.29	20.98	0.412
	2nd year	0.83	0.16	4.16	0.816
	3rd year	1.35	0.30	6.14	0.700
	4th year	0.92	0.27	3.21	0.900
Last-semester GPA	One point increase	0.75	0.33	1.72	0.501
Depression	One unit increase	1.26	1.13	1.41	**<0.001**
Mindfulness	One unit increase	0.95	0.93	0.98	**0.002**

**Table 4:** Multiple logistic regression for predictors of depression.

		Response depression = yes			
Covariate	Level	Odds ratio	95%CI low	95%CI up	OR P-value
Age (years)	One year increase	0.84	0.53	1.32	0.445
Gender	male	1.17	0.56	2.42	0.675
Academic year	1st year	1.59	0.23	11.21	0.640
	2nd year	0.77	0.16	3.72	0.742
	3rd year	1.40	0.32	6.13	0.658
	4th year	0.44	0.14	1.44	0.176
Last-semester GPA	One point increase	0.72	0.34	1.52	0.383
Personal history of meditation	Meditating	0.93	0.46	1.89	0.840
Stress	One unit increase	1.26	1.17	1.36	**<0.001**
Mindfulness	One unit increase	0.93	0.90	0.96	**<0.001**

### Moderation effect of mindfulness and its subscales on the impact of stress on depression

**Fig 2:**
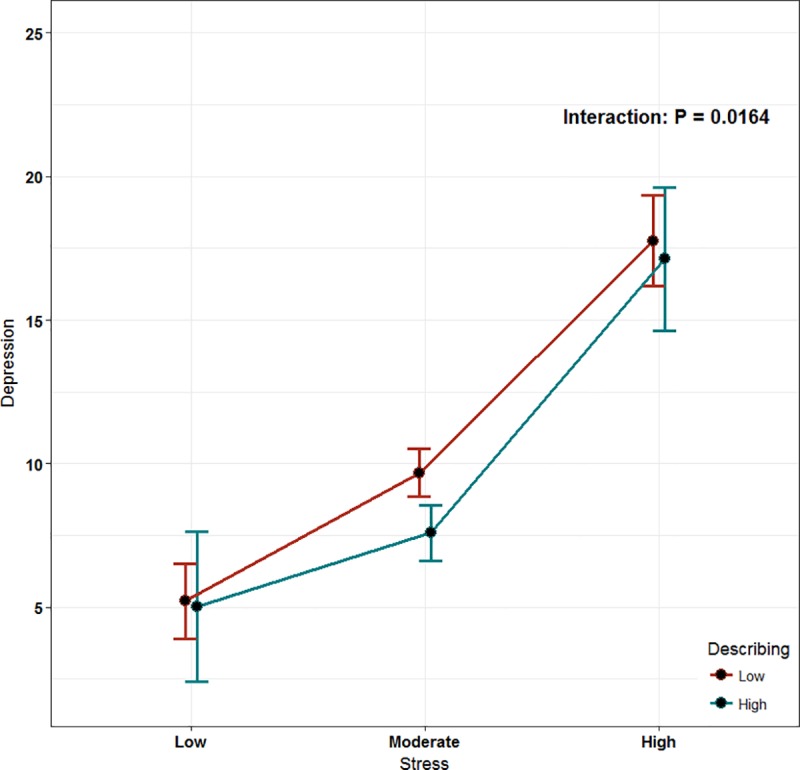
The effect of mindfulness (describing) as a moderator of stress.

**Fig 3:**
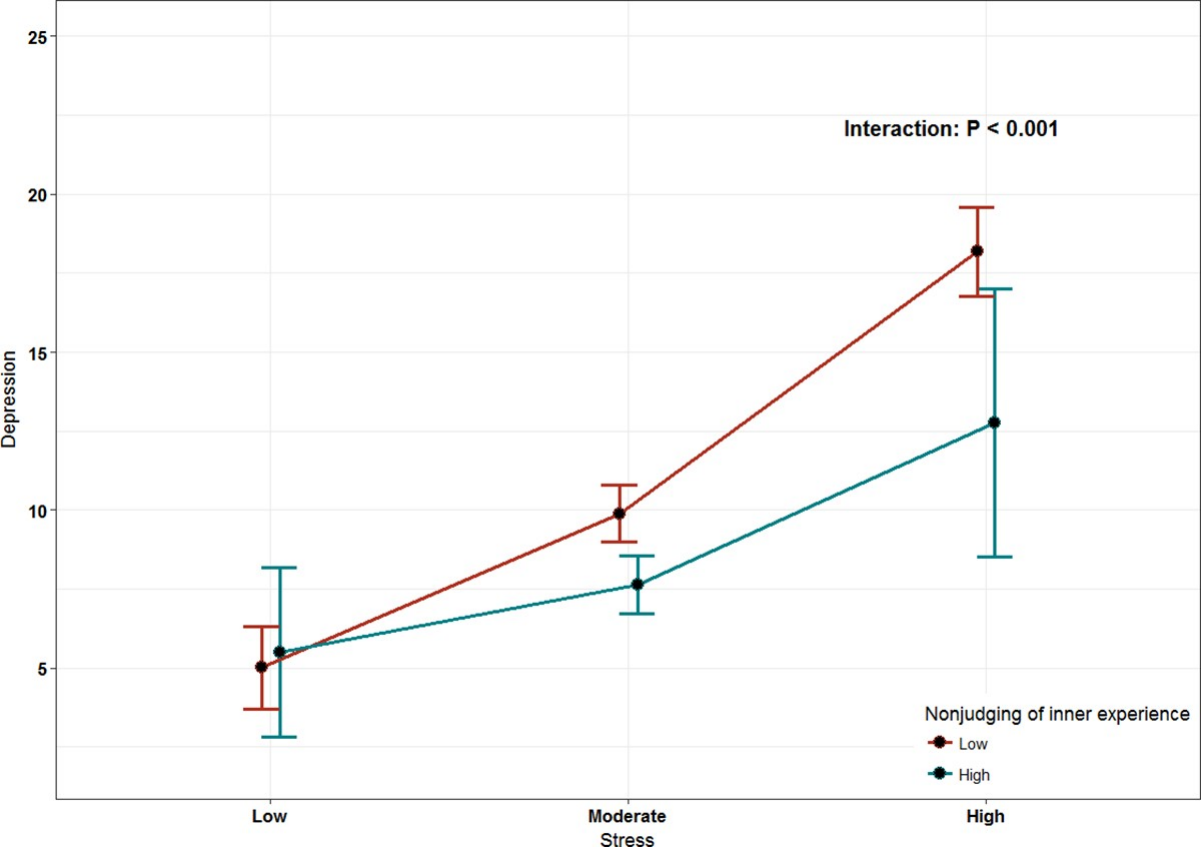
The effect of mindfulness (non-judging of inner experience) as a moderator of stress.

**Fig 4:**
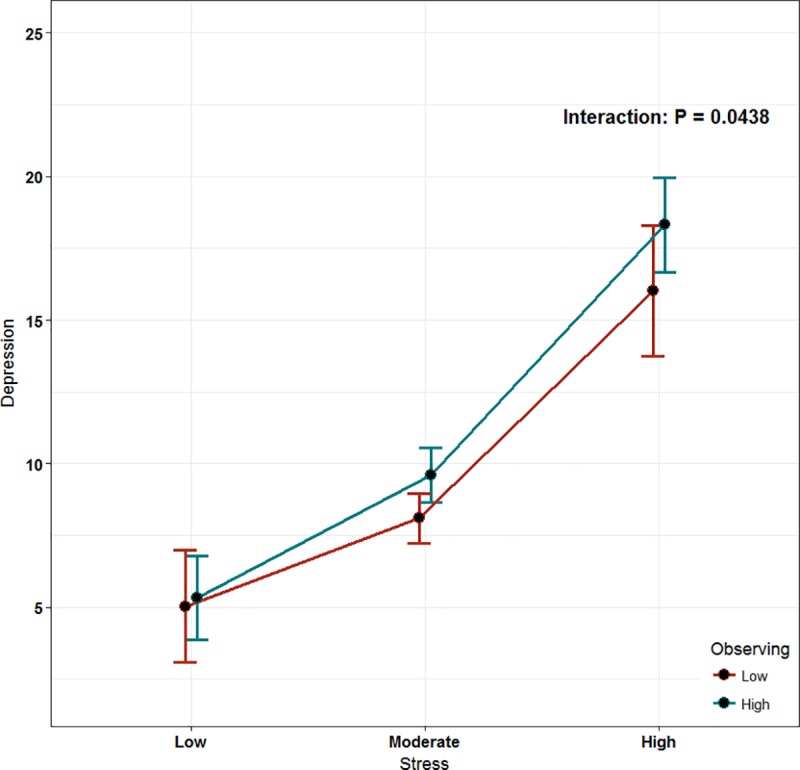
The effect of mindfulness (observing) as a moderator of stress.

The least-square means of interactions were used in this model to assess the moderation role of mindfulness. The mindfulness score was categorized into low and high based on our sample median. Only two subscales of mindfulness were found to play an inverse moderator role: describing and non-judging of inner experience (P = 0.0164, P < 0.001, respectively; Figs 2 and 3). However, the observing subscale was found to have a direct proportional moderating role (P < 0.0438, Fig 4).

## Discussion

The high prevalence of stress and depression among medical students of our study largely potentiates and supports the local and international data established previously [1–5]. These studies showed a high prevalence of impaired psychological wellbeing, namely stress and depression. However, the findings were distributed differentially on the spectrum of the intensity and magnitude of interest in the following two larger studies. A systematic review gathered information about studies reporting on stress and depression among medical students in the USA and Canada. The results revealed that both stress and depression were significantly higher than in the general population [1]. The second largest and fairly recent study looked at global publications on the prevalence of depression among medical students, which was found to be 28%. In addition, among all geographic regions, Middle East was found to have the highest prevalence at 31.8% [2].

These studies show overall increases in stress and depression, which align with our findings. Clearly, this is a conspicuous sign calling for a serious follow-up in the context presented by our study and two other local studies [4,5]. Indeed, it is a great concern in the field of medical education given its’ negative reflection on the mental well-being of future medical professionals, which could potentially affect the overall wellness of the community [6–8].

This study revealed that the average total mindfulness score was 118.84 (SD 15.60), which lies at the mid-point of the FFMQ score (39–195). This is comparable with community data published in a FFMQ validation study [25]. Therefore, it seems that being in medical school has no advantage in terms of being protected against or adaptive to mental distress in the context of mindfulness compared with the general population.

The negative relationship of mindfulness with stress and depression in our sample supports the findings in multiple studies of other populations [17, 19, 28]. A Canadian study of 135 first-year university students found that mindfulness was inversely related to stress with a correlation coefficient of -0.222 (P<0.05) [19]. Moreover, a population-based study assessing the association of mindfulness with mental health found that four subscales of mindfulness were negatively related to depression [28]. This revelation underscores the need to understand the exact mechanism by which mindfulness causes this phenomenon. The proposed mechanisms are a decrease in rumination and worry and an increase in positive reappraisal [29, 30]. Intention and attitude are the key components of mindfulness according to Shapiro et al. [31], which work through four mechanisms: self-regulation; emotional, cognitive, and behavioral flexibility; value clarification; and exposure.

We avoided using cumulative GPA to eliminate possible underestimation of senior-year students’ performance compared with juniors. Instead, we employed the last-semester GPA as an indicator of academic achievement. We did not find an association between GPA and other variables, which could be explained by the fact that the range of GPA is too small to detect any significant association.

We utilized a moderation model that is recognized as a suitable method to assess complex variable relationships. We explored the interaction of mindfulness with stress as a predictor of depression. Apparently, having high scores on the subscales of non-judging of inner experience and describing had a lowering effect on depressive symptoms in our highly stressed students. Similarly, a Swedish study investigated the interaction of different mindfulness subscales with multiple psychological outcomes, including depression and stress, but in the general population. However, they found that all mindfulness subscales except the observing subscale had a significant effect on depression through the moderation of perceived stress [28].

In contrast, we found that the observing subscale had a significant direct proportional moderator role, which is not favorable in this instance in that it increased depression. Theoretically, it has been suggested that the design of the observing subscale inherently cannot formulate patterns seen with other subscales, especially in a non-meditating population [25]. Our population mostly did not practice meditation (72%). Thus, such findings could be explained by the subjects being observers in a non-mindful manner, resulting in a negative psychological impact [25].

### Limitations

Our observational study design inherently lacks the ability to establish a cause-and-effect relationship between the assessed variables and is unable to control for unknown confounders. Moreover, our choice of target population poses a challenge in generalizing the findings to beyond the context of medical education. However, since a stratified random sampling technique was employed, the findings may be deemed useful in any giving setting of local medical colleges for subjects with similar characteristics.

### Recommendations

Instead of GPA, we recommend utilizing other indicators such as cognitive tasks, which could provide a better view of how mindfulness may relate to academic performance. We also aspire to encourage local researchers to test mindfulness as an amenable state in an experimental study to delineate the causality association with other variables, as well as to assess the effectiveness of mindfulness training on improving mental wellbeing in our community. Furthermore, we encourage local researchers to assess both positive and negative psychological outcomes in relation to mindfulness.

## Conclusion

Mindfulness was found to be associated with lower depressive symptoms and stress in medical students. Furthermore, in a stressful environment, being mindful may protect from depression. Overall, the results confirm the medical literature on this topic. This study could also serve as an enrichment for local studies as one of the first studies to address this subject in our area.

## Supporting information

S1 File The research data. (XLSX)
